# 

**Published:** 1875-01

**Authors:** 


### CONTENTS.

PAGE.

**CONTRIBUTIONS.**

Coming Exodus of Trade. BEN. H. PRATT............ 303
Healthfulness of Woolens. P. H. HAYES, M. D..... 305
Patent Medicines. M. S. BIDWELL....................... 307

**CLIPPINGS.**

Influence of Marriage Upon Health...................... 308

**OUR CORRESPONDENCE.**

Seven Letters with answers.................................. 316

**MEDICAL ITEMS AND NEWS.**

New Symptom of Bromism — Suppositories of Chloral — Erysipelas — Good Anodine — Eczema Papulosum — Tincture of Phosphorus — Phagedena — Sign of Pertussis — Chronic Diarrhœa — Burns — Night Sweats — Chronic Cystitis — Sick Headache — Tonic Prescription — Chilblains and Coryza -- Toothache -- Hæmorrhoids -- Use of Ergot -- Bronchitis -- Catarrh of Bladder -- Pruritus Vulvæ -- Purtussis -- Glycerine Jelly -- Hydatids -- Pneumonia and Bronchitis -- Eczema Capitis -- Rabies -- Colors for Show Bottles -- Delerium Tremens -- Diarrhœa of Phthisis -- Pleurisy -- Syphilitic Lichen -- Night Sweats of Phthisis -- Facial Erysipelas -- Internal Hemorrhage -- Neuralgia -- Treatment of Phthisic -- Hay Fever -- Hydrate of Chloral -- Chest Affections..............309 to 315

**HOUSEHOLD HINTS AND RECIPES.**

Polishing Stoves — To Render Carmine Brilliant — Paint for Wood — To fasten Handles &c. — Hardening Small Tools — New Cement — Cement for Marble — Cement for Metals and Glass — Improved Starch — To Soften Putty — Nutritive Meat Jelly — Liquid Glues — Rosewood Stain — Colored Ink — Simple Plan of Ventilation — New Method for Preserving Grapes..............317 to 319

**EDITORIAL.**

New Postal Law.............................................. 320
Cleft Palate.................................................... 320
The Cornea..................................................... 321
Deformed Feet................................................ 322

**EDITORIAL CHIT-CHAT.**

Five Cents Extra — Snow — Wonderful Lake -- Doctor Luke -- The Centennial -- Only a flower -- Plate Glass............................................................ 324
Index.............................................................. 327

